# FoodSwitch: A Mobile Phone App to Enable Consumers to Make Healthier Food Choices and Crowdsourcing of National Food Composition Data

**DOI:** 10.2196/mhealth.3230

**Published:** 2014-08-21

**Authors:** Elizabeth Dunford, Helen Trevena, Chester Goodsell, Ka Hung Ng, Jacqui Webster, Audra Millis, Stan Goldstein, Orla Hugueniot, Bruce Neal

**Affiliations:** ^1^The George Institute for Global HealthFood Policy DivisionCamperdownAustralia; ^2^University of SydneySydneyAustralia; ^3^Xyris SoftwareBrisbaneAustralia; ^4^Bupa AustraliaSydneyAustralia; ^5^Key to NutritionSydneyAustralia

**Keywords:** smartphone technology, traffic light labeling, food choices, public health nutrition, processed food

## Abstract

**Background:**

Front-of-pack nutrition labeling (FoPL) schemes can help consumers understand the nutritional content of foods and may aid healthier food choices. However, most packaged foods in Australia carry no easily interpretable FoPL, and no standard FoPL system has yet been mandated. About two thirds of Australians now own a smartphone.

**Objective:**

We sought to develop a mobile phone app that would provide consumers with easy-to-understand nutrition information and support the selection of healthier choices when shopping for food.

**Methods:**

An existing branded food database including 17,000 Australian packaged foods underpinned the project. An iterative process of development, review, and testing was undertaken to define a user interface that could deliver nutritional information. A parallel process identified the best approach to rank foods based on nutritional content, so that healthier alternative products could be recommended.

**Results:**

Barcode scanning technology was identified as the optimal mechanism for interaction of the mobile phone with the food database. Traffic light labels were chosen as the preferred format for presenting nutritional information, and the Food Standards Australia New Zealand nutrient profiling method as the best strategy for identifying healthier products. The resulting FoodSwitch mobile phone app was launched in Australia in January 2012 and was downloaded by about 400,000 users in the first 18 months. FoodSwitch has maintained a 4-plus star rating, and more than 2000 users have provided feedback about the functionality. Nutritional information for more than 30,000 additional products has been obtained from users through a crowdsourcing function integrated within the app.

**Conclusions:**

FoodSwitch has empowered Australian consumers seeking to make better food choices. In parallel, the huge volume of crowdsourced data has provided a novel means for low-cost, real-time tracking of the nutritional composition of Australian foods. There appears to be significant opportunity for this approach in many other countries.

##  Introduction

Growing rates of overweight and obesity around the world, in conjunction with a rise in nutrition-related diseases [1,2], mean that the food industry has an increasingly important role to play in public health. In high-income countries, the majority of food eaten is processed or pre-prepared by the food manufacturing, food retail, and catering industries [3]. These industries and their associated distribution networks have enabled a constant supply of affordable food in much of the world with consequent alleviation of many nutritional deficiency disorders [4]. However, a large proportion of the world’s population is now exposed to foods that are excessively energy dense and high in saturated fat, sugar, and salt [5,6]. Higher intakes of energy, saturated fat, added sugars and salt, such as that provided by a typical Western-style diet through a higher intake of processed food products, are risk factors for chronic disease, particularly cardiovascular disease, type 2 diabetes, and obesity [7-9].

It is difficult for consumers to make healthy food choices because the nutritional information available on packaging is not easily interpretable [10]. While packaged food in Australia is required to display a nutrient declaration [11], the format of the information provided can make it difficult for consumers to use this to make a quick and easy assessment of how healthy a product is [12]. It has been suggested that interpretive front-of-pack nutrition labeling systems have the potential to help consumers choose products on the basis of healthiness both by enabling an understanding of the nutrient data and allowing direct comparison across products [12]. There is widespread support from public health organizations for front-of-pack nutrition labels that provide “at a glance” information that consumers can act on [13]. This is based on evidence indicating that front-of-pack labels (FoPL) are much better understood than the nutrient declaration, particularly among disadvantaged groups who have the most to gain from making better decisions about the foods they buy [12]. There is, however, currently no requirement for FoPL in Australia. Australian industry has implemented “daily intake guides” [14] on some products, but market penetration is limited and the absence of any interpretive element is likely a major weakness [10]. It has been suggested that multicolored traffic light labels could be a better option for informing Australian consumers [10]. The Australian government is also currently exploring the use of a “star” system to rate products in terms of healthiness. However, this has not yet been implemented and industry is resisting the work program.

Mobile phone technologies offer an innovative way for consumers to access all manner of information. Mobile phones have become widely available in Australia over the last few years with more than 65% of Australians aged 15-65 years (8.6 million people) now owning some form of smartphone. This is a higher proportion of the population than in the United States and Britain [15]. In Australia, 90% of all smartphones use either an iOS or Android operating system, and 76% of Australian smartphone owners currently use their phones to get recommendations for health and other lifestyle-related factors [16]. A number of mobile phone apps have used the traffic light signposting system, but most have required the user to manually enter nutritional information about the product.

In the absence of standardized FoPL on Australian foods, and with processed foods largely responsible for excessive intakes of saturated fat, energy, added sugars, and sodium in the diet, we sought to develop a mobile phone app that would provide Australian consumers with access to easy-to-understand nutritional information about packaged and processed foods. To this end, we began a program of research and development to define how to use evolving mobile phone technology to deliver Australian consumers with better information about the packaged foods they eat. We intended that this project would ultimately improve the healthiness of the food choices made by Australian consumers and therefore enhance the health of the Australian population.

## Methods

### Objective

This project was planned and executed as a collaboration between researchers based at The George Institute for Global Health, developers from Xyris Software Pty Ltd, and the health communications team at Bupa Australia. The objective was to deploy a mobile phone app that would allow users to access quick and easy-to-understand information about the nutritional characteristics of packaged foods and, where possible, would suggest healthier alternative products.

### Identification of Required Nutritional Information

Packaged foods in Australia are required to display a Nutrition Information Panel (NIP) that includes information about seven key nutrients. The information in the NIP is known to be mostly accurate [17], and these label data were identified as the most likely source of comprehensive nutritional information to support the proposed mobile phone app. It was recognized, however, that food composition tables that collate this information at the level of the stock keeping unit (SKU) are scarce, and those that do exist are owned by commercial entities and can be expensive to access. In addition, we noted that a food categorization system that could divide food into comparable subsets of products would be required. A search was done using the Internet and by inquiry among colleagues to identify possible data sources. We also noted that the universal product code (UPC) (barcode) values for each SKU would be required for the delivery of an app based on barcode scanning, and thus the search was broadened to also capture databases of Australian UPCs.

### Selection of Display Format for Nutritional Information

There are a broad range of different formats that have been proposed for FoPL systems [12], and these were identified through a review of the literature. A series of criteria were developed to support the format that would be selected for the app based primarily on (1) the quality of the supportive evidence, (2) the likely acceptance of the format by consumers, (3) current FoPL practices around the world, and (4) congruence with local Australian standards and recommendations.

### Method of Product Comparison and Identification of Healthier Products

This project sought both to provide easily understandable nutritional information and to support consumers in their efforts to make healthier food choices. To better deliver on the second of these objectives, it was determined that it might be necessary to compare products against one another and develop a method of suggesting alternative products that were likely to be healthier. Fundamental to this requirement was a mechanism for ranking foods on the basis of their healthiness, and a review was undertaken to identify the different methods to achieve this. Once again, a series of criteria were considered in making the choice and these were based primarily on (1) the quality of the supportive evidence, (2) the likely acceptance of the method by the nutrition community, (3) current methods being used around the world, and (4) congruence with local Australian standards and recommendations.

### Choice of the Technological Solution

The project was launched with the goal of developing a mobile phone app. While we recognized early on that aspects of the solution might subsequently be deployed on other platforms, the focus of the work program was on the development of a mobile phone app for Australia. The key considerations in developing the technological solution were the method of user interaction with the mobile phone app and the platforms the app would be developed for. In regard to the user interaction, decisions were informed primarily by reviewing the functionality of other mobile phone apps—this review covered apps available in the broader retail space as well as those addressing specific health and nutrition issues. We chose the platforms the application should be developed for based on an analysis of the current and projected market penetration of the iOS, Android, Windows, and BlackBerry, in Australia.

### The Mobile Phone App Development Process

Development of the mobile phone app was led by individuals who are expert in research and public health, ensuring that the solution was underpinned by strong science and retained a primary public good objective. The findings of the reviews done during the development process were, however, reviewed by all participating groups (research, public health, developer, and health insurer) to ensure that decisions were informed by a broad range of perspectives. The development process was broken down into key components (eg, user interface, supporting data systems, and nutritional algorithms) that were formulated in parallel by working groups that were expert in the respective area. Weekly meetings were held to review progress and iteratively refine each component of the work. An initial test version of the mobile phone app was built and deployed for user acceptability testing by a limited group of individuals associated with the development team. Once feedback had been incorporated, a subsequent iteration of the app was deployed to a broader group of external stakeholders for further evaluation. Ultimately a launch version was completed and placed in the public domain.

##  Results

**Figure 1:**
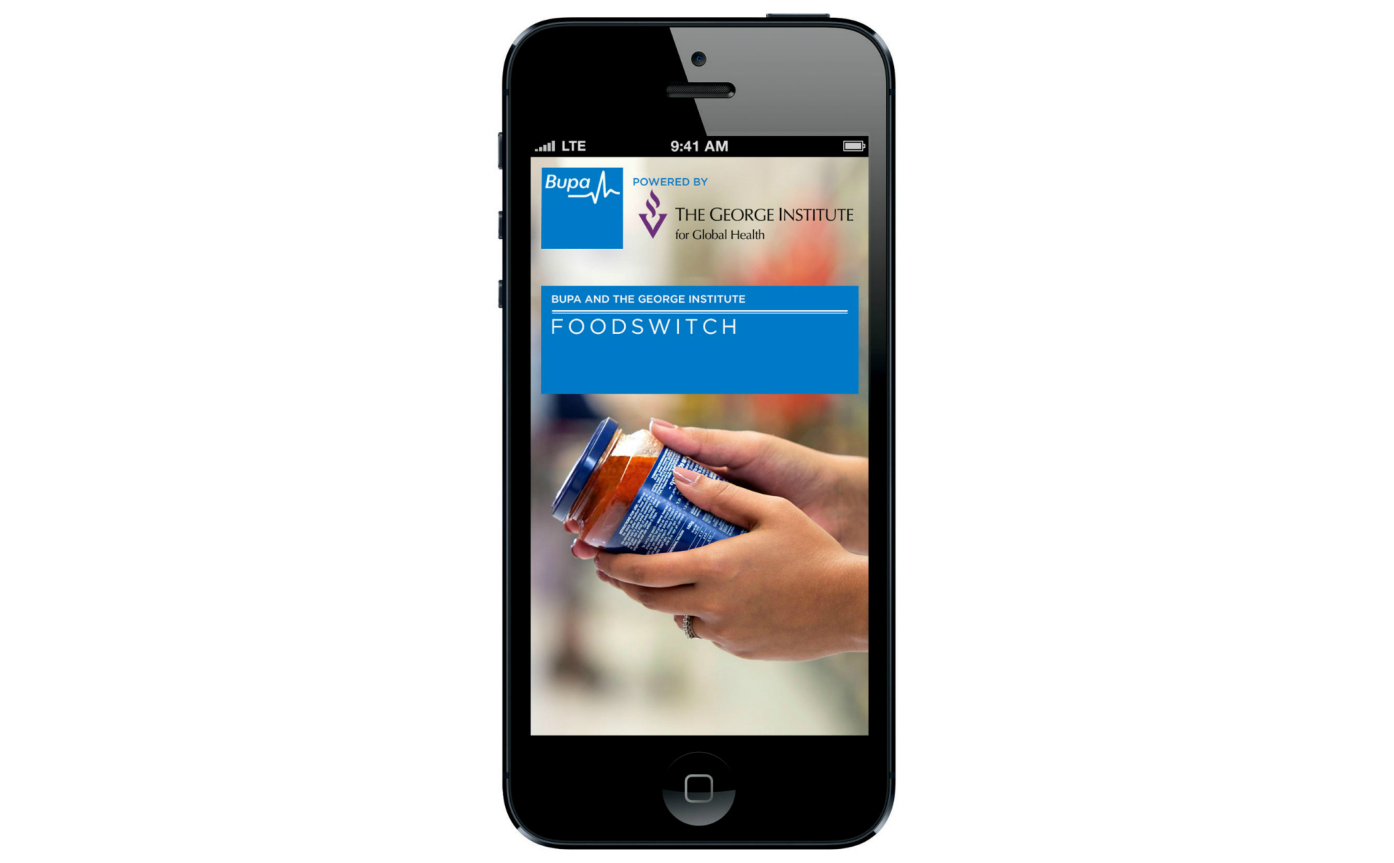
Splashscreen for FoodSwitch.

The development process commenced in August 2011, and the initial iOS version of FoodSwitch was deployed in January 2012 (Figure 1). This was followed by a launch on the Android platform in March 2012 with subsequent upgrades made to both platforms.

### Food Composition Database and Product Categorization

A branded food composition database compiled by The George Institute for Global Health and updated on an annual basis was identified as the source of nutritional information that was most complete and readily accessible for the FoodSwitch project. The 2012 database was compiled from in-store surveys done by George Institute staff at large stores in Sydney, Australia: ALDI, Coles, Independent Grocers of Australia, and Woolworths. During the surveys, the nutritional information provided on the NIP was transcribed into the database. A random selection of 5% of records was verified against the original NIP (by viewing product photos within the online database system). The 2012 dataset held records for about 17,000 SKUs and included nutrient information on energy, protein, total fat, saturated fat, carbohydrate, sugar, fiber, sodium, and calcium. The database also contained information on the UPC for each item. Each SKU in this database is assigned to one of about 700 different food categories, and this categorization system was adopted as the standard for FoodSwitch.

### Display Based on Traffic Light Labels

The review of possible display formats identified multiple colored traffic light labels as the preferred format. Using this approach, foods are signposted with respect to each of total fat, saturated fat, sugar, and salt using a high (red), medium (amber), or low (green) indicator in conjunction with a fifth circle without color containing information about energy density. The decision to select this approach was driven primarily by the report on the Australia and New Zealand Review of Food Labelling Law and Policy, “Labelling Logic”, which recommended that this system be adopted in Australia (Recommendation 51). The decision was further supported by the ready availability of detailed technical specifications provided by the UK Food Standards Agency in their 2007 document “Traffic light signpost labelling, Technical Guidance, Issue 2” [18]. This guidance details the method for front-of-pack traffic light signpost labeling including the thresholds for total fat, saturated fat, sugars, and salt at which red, amber, and green traffic light colors are applied for foods and beverages (Tables 1 and 2) [18].

**Table 1:** Food labeling.

	Green (low)	Amber (medium)	Red (high)	
Total fat	≤3.0 g/100 g	>3.0 to ≤20.0 g/100 g	>20.0 g/100 g	>21.0 g/portion
Saturated fat	≤1.5 g/100 g	>1.5 to ≤5.0 g/100 g	>5.0 g/100 g	>6.0 g/portion
Sugars	≤5.0 g/100 g	>5.0 to ≤12.5 g/100 g	>12.5 g/100 g	>15.0 g/portion
Salt	≤0.30 g/100 g	>0.30 to ≤1.50 g/100 g	>1.50 g/100 g	>2.40 g/portion

**Table 2:** Labeling for drinks.

	Green (low)	Amber (medium)	Red (high)
Total fat	≤1.5 g/100 ml	>1.5 to ≤10.0 g/100 ml	>10.0 g/100 ml
Saturated fat	≤0.75 g/100 ml	>0.75 to ≤2.5 g/100 ml	>2.5 g/100 ml
Sugars	≤2.5 g/100 ml	>2.5 to ≤6.3 g/100 ml	>6.3 g/100 ml
Salt	≤0.30 g/100 ml	>0.30 to ≤1.50g/100 ml	>1.50 g/100 ml

### Ranking by Nutrient Profile Score

The method selected to enable the comparison of food products was the nutrient profiling system (Nutrient Profiling Scoring Calculator - NPSC) developed by Food Standards Australia New Zealand (Textbox 1) [19]. This is an across-the-board scoring system where “baseline” points are allocated for increased amounts of energy, saturated fat, sodium, and total sugars [19]. These baseline points are then offset by “modifying” points allocated for the increasing percentage of the product that is fvnl (fruit/vegetables/nuts/legumes) and the amount of fiber, and for selected food categories, also protein and calcium. Foods are scored within three separate categories—edible oils, spreads, and certain cheeses; other foods; and beverages—with lower values indicating healthier products. Some scoring criteria (such as fiber and calcium) are not required on the standard Australian nutrient declaration and were therefore not completely available in the food composition database, requiring imputation of proxy values for some products (Table 3). All foods in the database were assigned a nutrient profile score using this system (Textbox 1).

**Nutrient profiling method.**

Step 1: Determine the NPSC category of the food.

- 1: beverages
- 2: all food not in category 3
- 3: cheese and processed cheese >320 mg calcium/100 g, edible oil, edible oil spread, margarine, and butter

Step 2: Calculate baseline points.

- 0-10 for energy
- 0-30 for saturated fat
- 0-10 for sugars
- 0-30 for sodium
- calculate total baseline points = X + X + X + X

Step 3: Calculate modifying points.

Fruit and vegetable (V points) (0, 1 ,2 , 5, or 8)

Formula used: (% non-concentrated fvnl) + (2 x % concentrated fruit or vegetables) ÷ (% non-concentrated fvnl) + (2 x % concentrated fruit or vegetables) + (% non fvnl ingredient) X 100/1

Protein points (P points)

- Calculate protein points (1-5)

Fiber points (F points)

- Calculate fiber points (1-2)

Step 4: Calculate the final score.

Final score = Baseline points – (V points) – (P points) – (F points)

**Table 3:** Data availability and imputation for nutrient profile scoring.

Nutrient	Availability
Energy	Complete
Protein	Complete
Total sugar	Complete
Saturated fat	Complete
Sodium	Complete
Dietary fiber	Partial data were available: products in a food category known not to contain fiber (eg, eggs) were assigned a fiber score of 0; products with data available were assigned an individual fiber score; and products with no data but in a category of foods known to contain fiber were assigned an imputed value. The imputed value was the average for all products in the category with data.
Calcium	Partial data were available for cheese and processed cheese categories, which require a calcium value for the calculation of the nutrient profile score: products with data available were assigned an individual calcium score, and products with missing data were assigned an imputed value. The imputed value was the average for all products in the category with data.
Percentage content of fruit, vegetables, nuts, and legumes (%F&V)	No data were available: products in food categories known not to contain appreciable amounts of fruit and vegetables (eg, dairy milk) were assigned a %F&V score of 0; and products in food categories known to contain fruit and vegetables were assigned imputed %F&V scores.

### Selection of the Technological Solution and Mobile Phone App Functionality

In Australia, the iOS and Android platforms comprise more than 90% of smartphones in circulation with only small numbers of users with Windows or BlackBerry platforms. The decision was therefore made to develop the mobile phone app only for the iOS and Android platforms. Initial development was done in iOS with subsequent transfer of the app to the Android environment. The transfer to Android involved moderate re-working of the user interface to achieve the best possible integration of the app into the Android environment.

**Figure 2:**
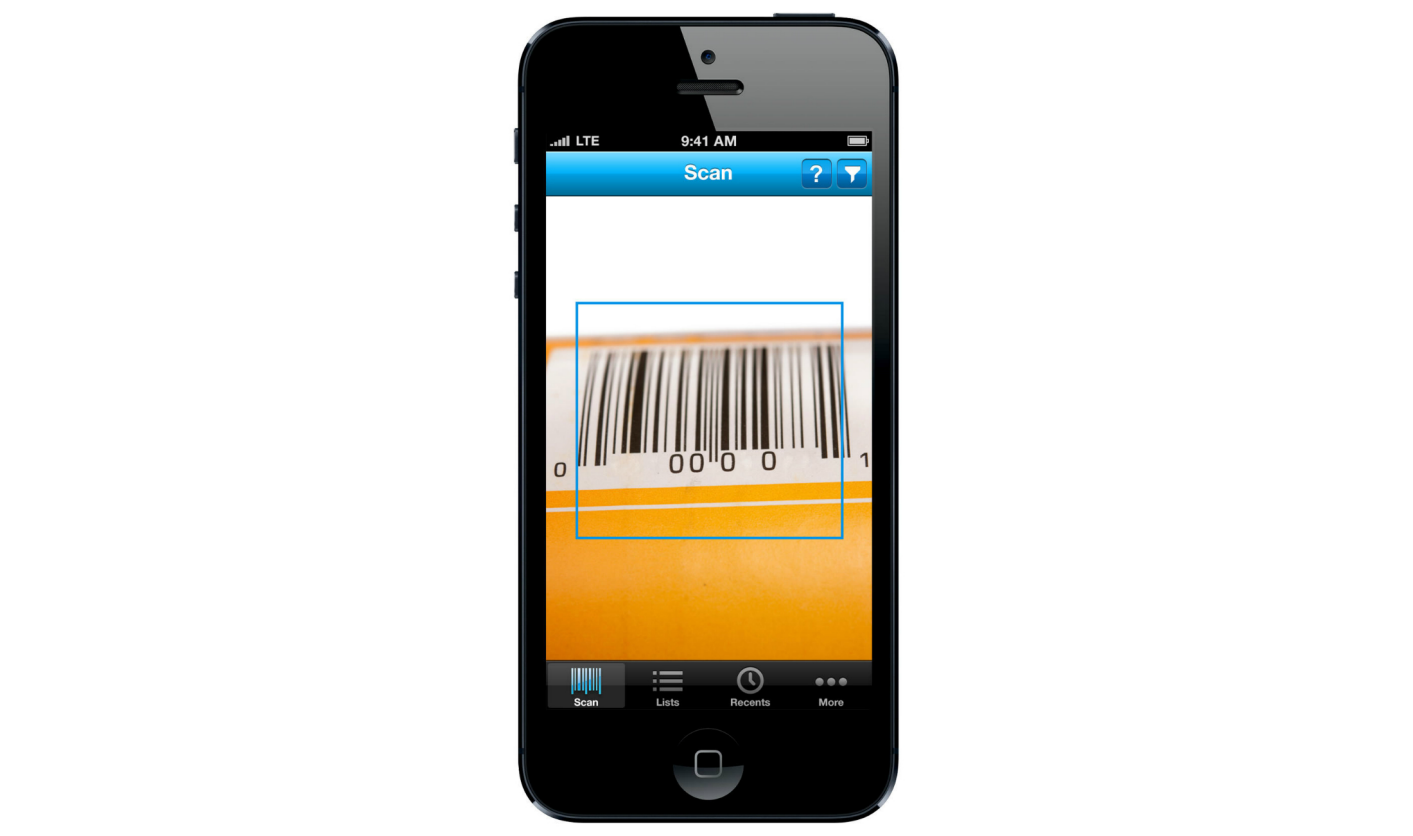
Scan function in FoodSwitch.

**Figure 3:**
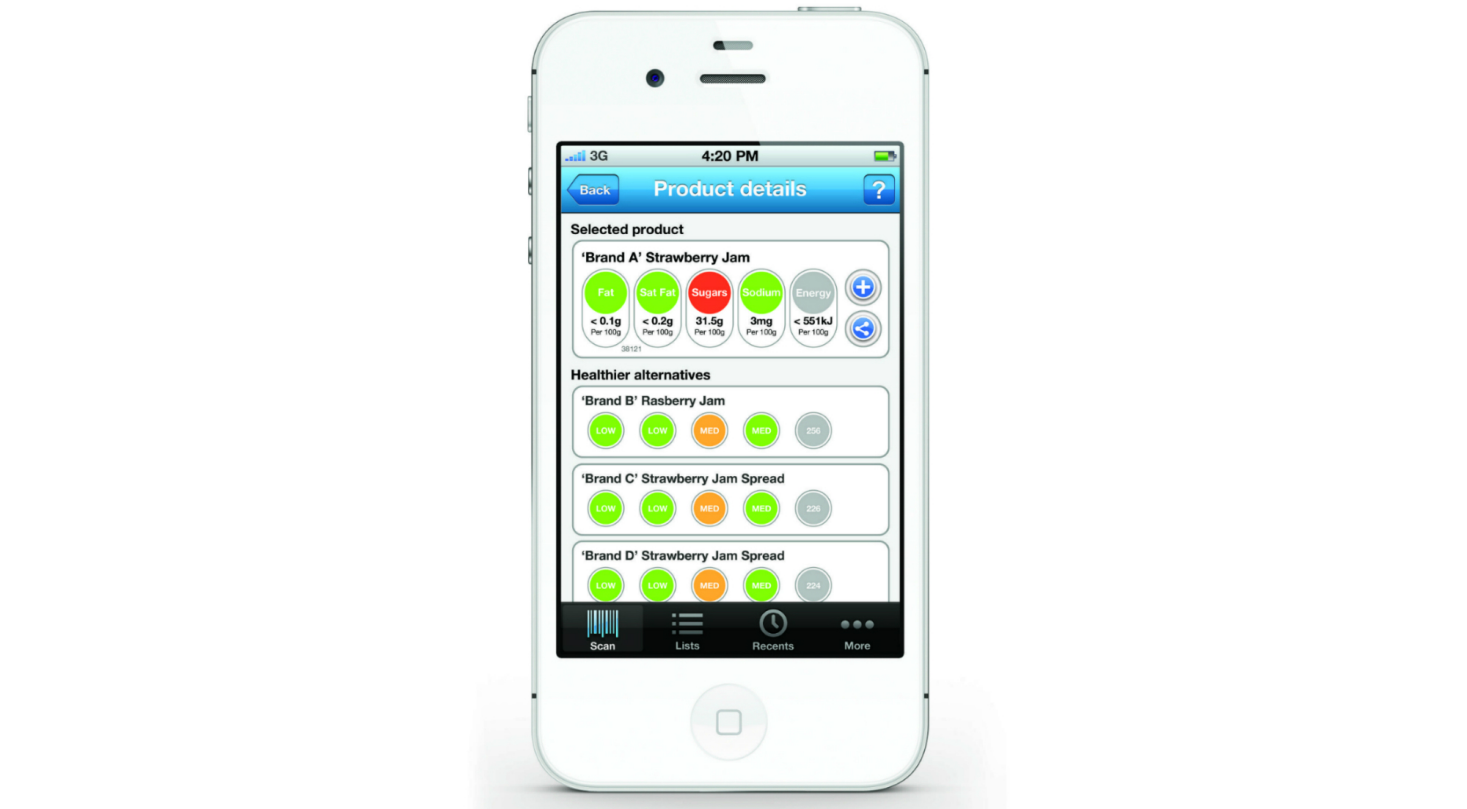
Switch function in FoodSwitch.

The over-riding design philosophy for the functionality of the app was simplicity of use and clarity of messaging. Accordingly, the primary operation of the app requires only that users turn it on and hold the mobile phone camera over the barcode of the product that they want information on (Figure 2). This action results in the automatic acquisition of the UPC, the immediate display of traffic light labels for the selected product, and the concurrent listing of similar alternative products with a more favorable nutrient profile (Figure 3). For some product categories where similar healthier alternative products are not recommended (such as sugar-sweetened soft drinks), a standard message provides high level advice about how to make a healthier choice (“Sugar-free drinks and water are healthier choices” in the case of sugar-sweetened soft drinks). Users are also able to compile lists, see the results of recent scans, and share their observations using social media. Multimedia Appendix 1 outlines the overall FoodSwitch process.

**Figure 4:**
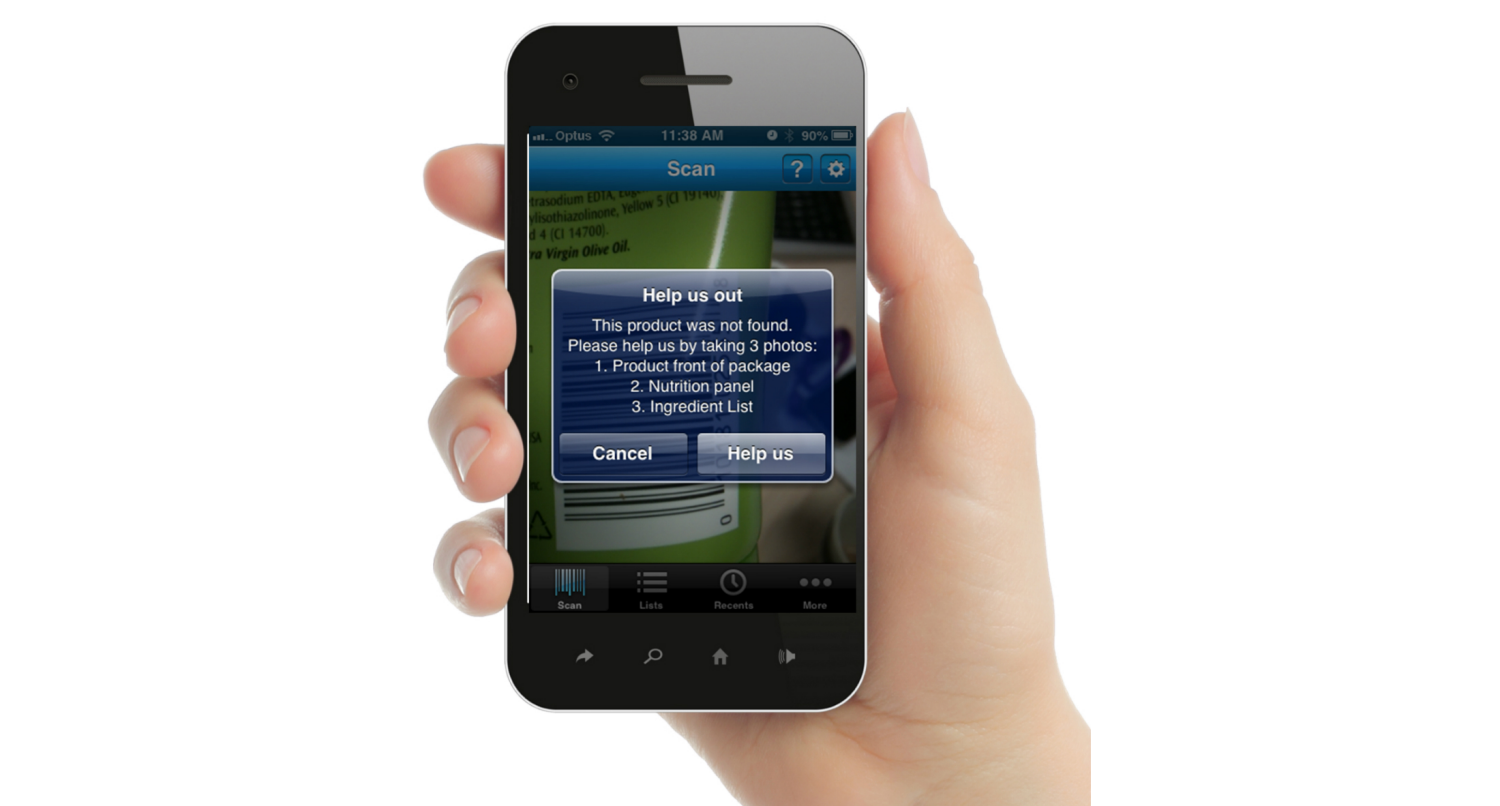
Crowdsourcing function in FoodSwitch.

A particular innovation in the app was the incorporation of a crowdsourcing function whereby users are able to contribute information on missing products. If a barcode is scanned but the corresponding UPC is not identified in the database, then the user is asked to photograph the front of the package, the NIP, and the ingredients list (Figure 4). The data are then forwarded to the data management center, and the information is added to the database. Periodic updates to the database are then made to ensure that the app is supported by complete and contemporary product information.

### Downloads, User Feedback, and Post-Launch Development

In the approximate 2-year interval between the launch of the FoodSwitch app in January 2012 and the preparation of this paper, FoodSwitch has been downloaded by more than 400,000 users. Some 2000 have provided comments, mostly positive, and the app has retained a 4+ score in the iTunes store since launch. In addition to updating the underlying food composition database, there have been two major upgrades that have added new functionality—SaltSwitch*,* targeted at individuals seeking to lower their dietary salt intake*,* which recommends healthier alternative products primarily on the basis of the sodium level, and GlutenSwitch*,* targeted at people with celiac disease and those wanting recommendations for gluten-free foods. Both developments were based on user feedback and a series of further upgrades that will address health problems such as diabetes and dyslipidemia are under development. The crowdsourcing tool within FoodSwitch has enabled substantial expansion of the underlying database from an initial 17,000 SKUs to over 50,000. More than 26,000 photographs, equating to 6000 additional SKUs, were received in the first 48 hours after launch, and 2 years later more than 200 photographs of new SKUs are still sent in by FoodSwitch users every day. While the initial photographs were mostly of products missing from the launch database, the photographs now received are of new items released into the market.

## Discussion

### Principal Results

The success of FoodSwitch with Australian consumers is likely a direct reflection of the enormous community interest in the foods they eat and the great difficulty they have in obtaining easy-to-understand nutritional information. The FoodSwitch experience provides strong support for the introduction of a standardized, interpretive front-of-pack food labeling system for all foods marketed in Australia. Last year, the Australian government indicated that it will launch a “star”-based FoPL system that will be mandated if widespread voluntary uptake by industry is not forthcoming. While data to define the impact of the proposed star-based system are sparse, the application of a single FoPL system across all foods is an important first step in providing consumers with the information they require to make healthier food choices. Plans are in place to provide users of FoodSwitch with the option to select the new star-based labeling system as the display mode, as soon as the criteria underpinning the system are finalized. Funds have also been received to conduct large-scale randomized trials using the FoodSwitch technology that will, for the first time, objectively define the impact of different labeling systems on real world food purchases.

The implementation of FoodSwitch in Australia was initially done with the objective of providing consumers with a tool that would enable them to make better food choices, both by providing nutritional information in an at-a-glance, easy-to-understand format and by suggesting healthier alternative products within the same category. The app has been successful in this regard with a large number of downloads and a significant number of regular users. However, almost all of the Australian population consumes a diet that is suboptimal in nutritional content, and even if every individual who downloaded FoodSwitch made radical changes to their diet, the net effect on population health would be only small. While this does not mean that FoodSwitch is without direct value to some users, the direct benefits are likely to be restricted to the households of a relatively few motivated individuals.

Fortunately, individual behavior change is not the only means by which FoodSwitch might have public health impact. Indeed, the real value of FoodSwitch is likely to be in the data collected through crowdsourcing and a consequent new capacity to define and track the nutritional composition of the food supply over time. These data will make it possible to hold the food industry to account, as a group and at an individual company level, for the nutritional quality of the products they sell. It will also be possible to objectively evaluate the extent to which the food industry and government deliver on commitments to improve the quality of the food supply through initiatives such as the Food and Health Dialogue. Another postulated effect of FoPL is that it will drive reformulation of products towards healthier formulations; for example, manufacturers might reduce the levels of adverse nutrients such that the product displays an amber instead of a red, or a green instead of an amber traffic light. It will be possible to directly test this hypothesis using the data collected by FoodSwitch in the coming years.

For the first few years of operation, compilation of the branded food composition database that FoodSwitch was based on a highly labor-intensive exercise that incurred significant costs, while achieving only partial coverage of the Australian processed food supply. By crowdsourcing data collection through FoodSwitch itself, the data collection costs have been optimized, the database is much more comprehensive, and new products are added soon after they appear on the supermarket shelves rather than during an annual round of data collection. With streamlined data management systems in place, and plans to develop optical character recognition for data entry and automated algorithmic categorization of foods, it should be possible to further reduce costs and enhance data processing. It is also hoped that this will provide for scalability and offer a practical means of delivering on the ambitions of organizations like the Global Food Monitoring Group [20] and INFORMAS [21], who seek to track the food supply in multiple countries around the world.

FoodSwitch was initially launched in Australia but is now also available in New Zealand and the UK, with well-developed plans for release in multiple other countries in the short-to-medium term. Each country requires its own database for the operation of FoodSwitch because UPC codes, product ranges, and the nutritional content of even apparently identical products vary from one jurisdiction to another. In practice, this means that the launch of FoodSwitch requires access to an initial “priming” branded food composition database for that country, which can then be made complete by post-launch crowdsourcing. The priming dataset needs to ensure an initial rate of successful scans that is sufficiently high to persuade early adopters of the value of submitting photographs of missing items. Just how high that successful scan rate must be is uncertain, although it is clear that the approximate 70% success rate achieved at launch in Australia was more than adequate. The potential for crowdsourcing data collection of the initial priming dataset is also under investigation through the engagement of special interest groups willing to submit photographs of items with the promise of a future launch of FoodSwitch in their country.

### Limitations

Even with mandated nutrient declarations on processed foods in Australia, access to the full data required for nutrient profiling was a challenge for the FoodSwitch project, with incomplete information available for calcium, fiber, and the fruit and vegetable content for many products. In each case, the missing values were imputed to enable a score to be calculated for each product. In several cases, this resulted in the same average value being applied to multiple products, which may have reduced the capacity to discriminate between the healthiness of the products within a given category. In particular, this resulted in little capacity for the fruit and vegetable content of a product to influence the ranking of products because the same score was applied to many products. Future iterations of FoodSwitch may make recommendations for healthier alternatives outside of category, in which case this limitation will be reduced. As with many databases of this scale, despite rigorous quality assurance being undertaken regularly on the data, errors may exist, either in data entry itself, or in the values displayed by the manufacturer on product labels. Within FoodSwitch*,* users are able to send feedback on data errors directly to the FoodSwitch team so that the next database update can include fixes to existing data. Another limitation is that nutrient values per serving of product are not displayed within FoodSwitch. However, there are plans for future versions of the app to display this information.

### Conclusions

FoodSwitch has greatly exceeded our expectations both in regard to numbers of downloads and the crowdsourcing of data. Based on this success and the clear potential for scalability, there are a series of further upgrades planned and we hope to launch the app with partners in multiple developed and developing countries around the world. The proposed model is one whereby the local partner organization takes leadership of the project in their country with the Australia-based team providing technical support and advice about how to achieve launch and the requirements for post-launch support. We hope that local partners will also take the lead in using the data that derive from the project to advocate for in-country improvements in the food supply, as well as contributing to the wider global effort to control the harms caused by the consumption of unhealthy processed foods.

## Multimedia Appendix 1

FoodSwitch process.

## Abbreviations

FoPL: front-of-pack labeling
fvnl: fruits, vegetables, nuts and legumes
NHMRC: National Health and Medical Research Council
NIP: nutrition information panel
NPSC: nutrient profiling scoring calculator
SKU: stock keeping unit
UPC: universal product code
