# Supplementary figures and images for: FoodSwitch: A Mobile Phone App to Enable Consumers to Make Healthier Food Choices and Crowdsourcing of National Food Composition Data
Source: JMIR Mhealth Uhealth. 2014 Aug 21;2(3):e37. doi: 10.2196/mhealth.3230 (PMC4147708; doi:10.2196/mhealth.3230)

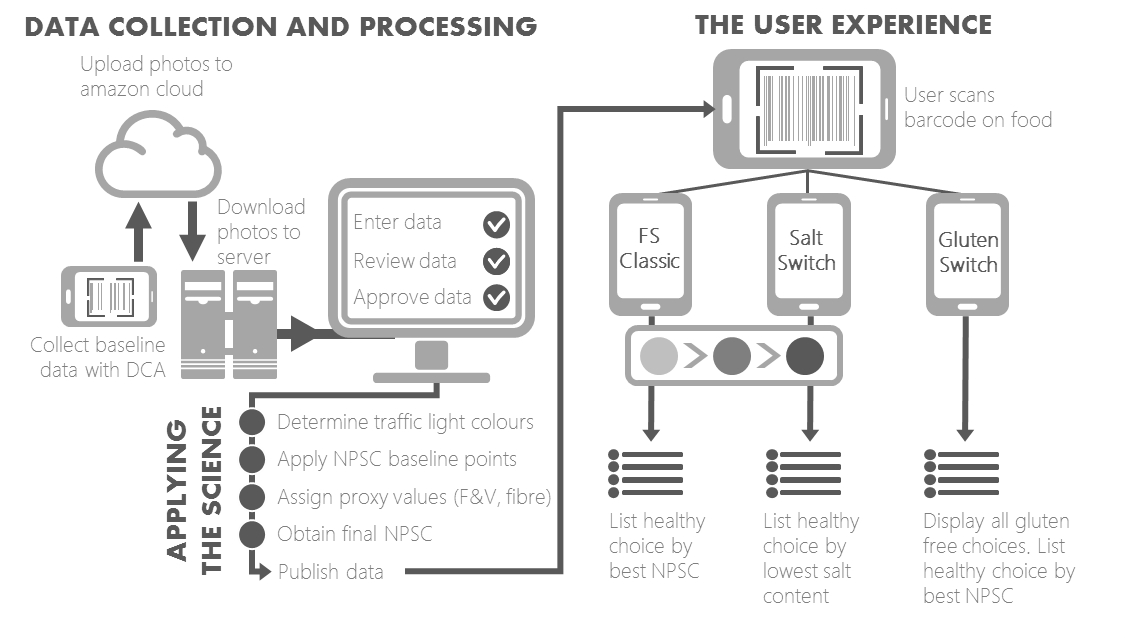

Supplement: Supplementary file 1 [file mhealth_v2i3e37_app1.jpg]
